# Phantosmia in Parkinson’s Disease: A Systematic Review of the Phenomenology of Olfactory Hallucinations

**DOI:** 10.3390/neurolint16010002

**Published:** 2023-12-22

**Authors:** Tommaso Ercoli, Caterina Francesca Bagella, Claudia Frau, Elisa Ruiu, Sabrine Othmani, Giansalvo Gusinu, Carla Masala, Leonardo Antonio Sechi, Paolo Solla, Giovanni Defazio

**Affiliations:** 1 Department of Neurology, University of Sassari, Viale S. Pietro 10, 07100 Sassari, Italy; ercolitommaso@me.com (T.E.); caterina.bagella@aouss.it (C.F.B.); clafrau@hotmail.it (C.F.); elisa.ruiu@aouss.it (E.R.); sabrine.othmani@hotmail.it (S.O.); 2 Department of Biomedical Sciences, Sassari University, 07100 Sassari, Italy; ggusinu@uniss.it (G.G.); sechila@uniss.it (L.A.S.); 3 Department of Biomedical Sciences, University of Cagliari, SP 8 Cittadella Universitaria, 09042 Monserrato, Italy; 4 Department of Translational Biomedicine and Neuroscience, University of Bari, 70121 Bari, Italy; giovanni.defazio@uniba.it

**Keywords:** Parkinson’s disease, non-visual hallucinations, olfactory hallucinations, phantosmia

## Abstract

Olfactory dysfunction is a prevalent non-motor symptom in Parkinson’s disease (PD), affecting approximately 65–90% of subjects. PD patients may also report odor perception in the absence of any external source, often referred to as olfactory hallucinations (OHs) or phantosmia. This study aims to explore the current understanding of OHs in PD and offer a comprehensive overview of their prevalence and characteristics. We conducted a systematic search of the literature published on PubMed from inception to July 2023 regarding OHs in PD, following PRISMA guidelines. From the 2875 studies identified through database searching, 29 studies fulfilled the necessary criteria and underwent data extraction. The frequency of OHs in PD patients varies widely, ranging from 0.5% to 18.2%, with female prevalence ranging from 36% to 75% of the patients. Olfactory experiences may vary widely, ranging from pleasant scents to unpleasant odors. Several studies have indicated the concurrent presence of other types of hallucinations alongside phantosmia, especially visual and auditory hallucinations. OHs in PD are a type of hallucination that has been largely overlooked. To gain a deeper understanding of OHs in PD patients, the next crucial step should involve the development and validation of a dedicated questionnaire.

## 1. Introduction

Parkinson’s disease (PD) is an increasingly prevalent neurodegenerative disorder characterized by motor symptoms that generally include bradykinesia, rigidity, tremor, and postural instability [1,2]. PD is becoming a significant source of disability and mortality among neurological conditions [3]. Considering recent epidemiological data, the estimated prevalence is at about 94 cases per 100,000 subjects, and it has been postulated that about 0.3% of the global population aged over 40 years old might be affected [3]. The annual incidence of new PD cases varies between 8 and 18.6 per 100,000 person-years [4]. Age is one of the most important risk factors, with the vast majority of subjects diagnosed at over 60 years old. Genetic predisposition may also play a role, as many gene mutations have been discovered thanks to modern techniques and have been linked to disease onset. This may explain the fact that individuals with a family history of PD are at a higher risk of developing the disease [5]. Other factors, such as dyspepsia, exposure to pesticides, oils, metals, and general anesthesia, have been associated with an increased risk of PD onset [5]. On the other hand, coffee consumption, smoking, and physical activity have been indicated as putative protective factors for the development of the disease [5].

PD may also manifest with a wide spectrum of non-motor symptoms (NMSs) as a result of the impairment of several neuronal systems, such as cognitive impairment, autonomic failure, smell and taste deficits, and sleep disorders [6,7]. Due to the relevant impact of these non-motor manifestations on the quality of life, it is crucial for neurologists to recognize and manage them alongside the cardinal motor symptoms of PD [6,7]. Cognitive disturbances are frequent among these NMSs, especially over the disease’s course; indeed, many PD patients experience issues with memory, attention, and executive functions. Mood disorders, such as depression, anxiety, and apathy, may further worsen the clinical picture, and sometimes they may even precede the onset of motor symptoms. Autonomic dysfunction is another common presentation of NMSs in PD patients, who may suffer, for instance, from orthostatic hypotension. Gastrointestinal issues like constipation can become chronic concerns, as can urinary problems, ranging from urgency to incontinence. Sleep disturbances in PD may present in several ways, such as insomnia or conditions like rapid eye movement sleep behavior disorder (RBD). Interestingly, some of these NMSs can manifest years before the cardinal motor signs and might be used as early markers of PD [6,7].

Within this context, olfactory dysfunction, one of the most common NMSs in PD, can be detected in approximately 65–90% of individuals with this condition [8,9]. Frequently, this symptom emerges before the presentation of cardinal motor signs and may be classified as a diminished or absent sense of smell (hyposmia or anosmia), altered smell perception (parosmia), or the experience of perceiving a smell without an external source, often referred to as olfactory hallucinations (OHs) [10,11]. Several methods may be used to assess olfactory dysfunction in Parkinson’s disease patients. The two most widely used are the University of Pennsylvania Smell Identification Test (UPSIT) and the Sniffin’ Sticks test. The UPSIT is a 40-item test built with different scratch and sniff strips integrated with microencapsulated odorant and designed to assess the ability to identify various scents [11]. The Sniffin’ Sticks test is a three-step assessment that evaluates different olfactory tasks, including odor identification, odor discrimination, and odor threshold. Based on the sum of these three values, known as the threshold–discrimination–identification (TDI) score, a patient can be categorized as normosmic, hyposmic, or anosmic [11].

OHs, or phantosmia, a term derived from the Ancient Greek words φάντασμα (ghost) and ὀσμή (smell), may occur frequently in PD patients, although they are often overlooked in clinical practice. OHs are perceptual disturbances where an individual experiences a smell that is not present in the environment [11]. These olfactory experiences may vary from pleasant (i.e., flowers and fruits) to unpleasant (i.e., rotten eggs, garbage, smoke) odors, with potential impacts on patients’ quality of life [11,12]. While OHs have been frequently studied in the context of psychiatric disorders or neurological conditions due to epilepsy or COVID-19 infection, their occurrence in PD is less understood [13,14,15].

This systematic review aims to explore the current knowledge on OHs in PD. Namely, it seeks to provide a comprehensive overview of the prevalence and characteristics of OHs in PD. We have specifically focused our attention on the frequency of OHs in the PD population and the gender distribution; the features of phantosmia (pleasant vs. unpleasant); the assessment methods used by the authors in order to evaluate OHs in PD patients; the olfactory function of the PD patients with phantosmia; the associated hallucinations that might coexist with OHs in the same subject; the impact of phantosmia on the quality of life of PD patients; and the treatment used to manage phantosmia.

## 2. Methods

We conducted a systematic search of the literature published on PubMed from inception to July 2023 using the following searching string: (((((phantosmia) OR (phantom smell)) OR (ghost smell)) OR (imaginary odor)) OR (olfactory hallucination)) AND (Parkinson*). We performed a systematic review following the Preferred Reporting Items for Systematic Reviews and Meta-Analyses (PRISMA) guidelines. These guidelines are globally recognized for providing consistency and transparency when researchers want to write a systematic review. By adhering to PRISMA, we aimed to provide a robust and replicable methodology, aiming to accurately represent the current state of knowledge on phantosmia in PD patients. The study protocol was submitted on the International Prospective Register of Systematic Reviews. The reference list of each selected article was checked to screen for additional studies possibly worth including that had not been captured by the original search method. Only original studies published in English in peer-reviewed journals were selected, and unpublished data, such as conference abstracts, were not considered.

After duplicates were removed, the title, abstract, and keywords of the retrieved publications were screened by TE, and irrelevant studies were excluded. Full-text papers were then independently reviewed by TE and PS for eligibility and included if they comprised case reports/series or studies investigating OHs in PD, either alone or in combination with other kinds of hallucinations, including minor hallucinations (i.e., sense of presence, passage, and illusions), visual hallucinations, tactile hallucinations, auditory hallucinations, and gustatory hallucinations. Longitudinal studies were included only if the baseline data were relevant to the purpose of this review. Differences in study selection between TE and PS were resolved by consulting with another author.

## 3. Results

**Figure 1:**
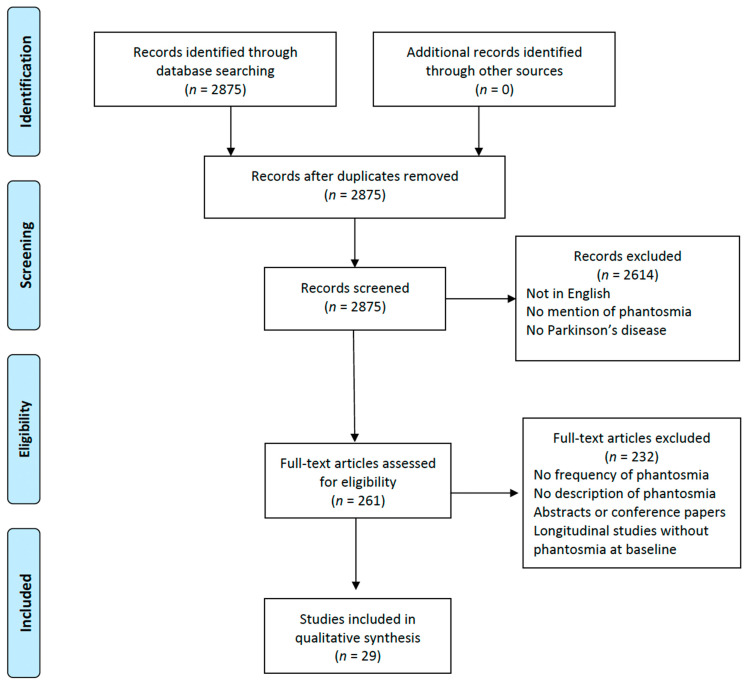
Study flow chart depicting the phases of this systematic review according to the Preferred Reporting Items for Systematic Reviews and Meta-analyses (PRISMA) flow diagram guidelines.

From the initial pool of 2875 studies identified through our comprehensive database searching, 2614 records were excluded. The primary reason for their exclusion was that either their titles or abstracts did not resonate with the central aim of this review. Our main focus was to investigate the occurrence and implications of OHs in patients with PD. Many of the excluded studies fell short of our criteria, such as being published in a language other than English, making no reference to phantosmia, or not including Parkinson’s disease patients in their study group. This rigorous screening ensured that only the most relevant articles were considered for further examination in our review. After assessing 261 full papers for eligibility, only 29 studies fulfilled the necessary criteria and underwent data extraction (Figure 1) [16,17,18,19,20,21,22,23,24,25,26,27,28,29,30,31,32,33,34,35,36,37,38,39,40,41,42,43,44]. Among the 232 full-text articles excluded, none met the inclusion criteria. This was either because the studies did not assess the frequency of phantosmia, lacked descriptions of OHs, were abstracts or conference papers, or were longitudinal studies that did not describe phantosmia at baseline. The information extracted from the selected studies, including the year of publication, study design, sample size, and number of patients with OHs, is reported in Table 1. Overall, we included 203 PD patients with phantosmia from the 29 studies included in this review. Due to the significant heterogeneity among the aforementioned studies in terms of population, methodology, and assessment of OHs, the data were framed into a narrative review, covering the frequency of OHs in the PD population and the gender distribution; the characteristics of phantosmia; the assessment methods used by the researchers in order to evaluate OHs in PD patients; the olfactory function of the PD patients with phantosmia; the associated hallucinations that might coexist with OHs in the same subject; the possible impact of phantosmia on the quality of life of PD patients; and the treatment used to manage phantosmia.

### 3.1. Frequency of OHs and Gender Distribution

As shown in Table 1, the frequency of OHs in PD patients varies widely, ranging from 0.5% to 18.2%, probably because of differences in the study population and methodology. Some studies listed in Table 1 are case reports; hence, the percentage indicating the presence of OHs for those investigations was not provided. A distinctive analysis was conducted by Aarsland and colleagues, who found no statistical differences in the prevalence of phantosmia between PD patients with and without dementia (6% vs. 1%, *p* = 0.14) [16]. Lenka and colleagues explored the occurrence of OHs in PD patients with psychosis and found that only one patient (1.9%) had phantosmia [28]. Conversely, baseline data from two double-blind studies of olanzapine for PD patients with drug-induced psychosis indicated a 16% frequency of OHs [21]. When considering only drug-naïve PD patients, Pagonabarraga et al. noted that 4% of their sample population reported OHs [36]. In the study by McAuley and Gregory involving 188 PD patients, 4 individuals indicated the presence of OHs (2.1%). Notably, over the subsequent three years, an additional two patients (1%) from this cohort, initially unaffected, developed phantosmia. Both consistently met the diagnostic standards for idiopathic PD during this span and showed no signs of cognitive impairment [32].

Considering the studies where the gender distribution of OHs in PD patients was available, we observed contrasting findings. Specifically, the studies by Solla et al. and Bannier et al. indicated a female prevalence of 75% and 67%, respectively [19,40]. However, in Mehanna’s study, women with phantosmia constituted 36% of the PD patients suffering from OHs [33]. When focusing solely on the patients described in the case or series reports, we found that seven women (54%) were affected by phantosmia [25,27,42].

### 3.2. OHs Features (Pleasant vs. Unpleasant)

OHs in PD patients have been described in reports with diverse characteristics. These olfactory experiences can vary widely, ranging from pleasant scents such as flowers and fruits to unpleasant odors like rotten eggs, garbage, and smoke.

A systematic assessment of phantosmia characteristics among PD patients was only available in four studies, and pleasant odor sensations predominated in three of them. For instance, in the study by Kulick and colleagues, 54.5% of the patients who experienced OHs described their misperceptions of smell as pleasant [26]. Moreover, in Kulick’s cohort, every individual who experienced olfactory hallucinations described perceiving strong, unpleasant scents. Four of these patients specifically identified these as offensive odors, like trash, combustion, or other harmful scents [26]. Mehanna and colleagues reported that 68% of PD patients with OHs described the features of their phantosmia as pleasant; however, the most commonly reported OH was that of smoke [33].

Among the 16 patients with phantosmia described by Solla et al., 13 (81.3%) reported pleasant smells such as flowers and fruits, while the remaining 3 patients (18.7%) described a perception of unpleasant scents such as garbage and rotten eggs [40].

Conversely, only two out of the nine (22.2%) PD patients with OHs described by Bannier and colleagues reported the perception of pleasant odors like flowers. In this particular sample, phantosmia predominantly manifested as unpleasant scents such as those of burned, spoiled, or rotten food or gas [19].

In the case reports/series data, 4 PD patients reported experiencing pleasant scents, whereas 13 PD patients indicated otherwise. Among those who perceived pleasant odors, one patient described the phantosmia as highly delightful, reminiscent of either “fine cuisine” or fruity fragrances, and exhibited a heightened olfactory acuity with an extended duration of scent perception upon exposure to odorant stimuli [25]. Another patient likened the experience to the pleasant aromas of a “rainy day” or even the scent of a “wet dog” [27]. The remaining two patients identified their phantosmia as having a perfume-like aroma [27,32].

Of the 13 PD patients who perceived unpleasant scents, the majority identified them as smoky or burning smells [25,32,36,42,43]. One patient likened the phantosmia to the aroma of oily and spicy foods [17], another described it as similar to “boot polish” [32], yet another related it to the scent of rotting fish [43], and one patient identified it as dog feces [32]. Two patients, in particular, associated their phantosmia with non-specific unpleasant odors emanating from their beds [18,32].

### 3.3. Assessment of OHs

The methods used to investigate the presence of phantosmia in PD patients varied among the studies included in this review. Considering the absence of a specific tool for assessing OHs in PD patients, most authors opted to interview patients using semi-structured questionnaires or non-specific questions related to the presence of phantosmia [16,18,19,25,27,28,29,32,33,35,42,43]. Other authors assessed phantosmia in PD patients using structured questionnaires or interviews, including the Neuropsychiatric Inventory (NPI), the Schedule for Assessment of Positive Symptoms (SAPS), the Enhanced Scale for Assessment of Positive Symptoms in Parkinson’s Disease (eSAPS-PD), the MDS-UPDRS hallucinations and psychosis item, the Psycho-Sensory hAllucinations Scale (PSAS), the Psychosis and Hallucinations Questionnaire (PsycH-Q), and the University of Miami Parkinson’s disease Hallucinations Questionnaire (UM-PDHQ) [17,20,21,22,23,24,26,30,31,34,36,37,38,39,40,41,44]. However, the available data in the literature did not identify the most appropriate tool to assess the presence of phantosmia in PD patients.

### 3.4. Quantitative Evaluation of Olfactory Function

Only five studies provided a quantitative evaluation of smell in PD patients with OHs. The Sniffin’ Sticks test was utilized in a large cohort by Solla and colleagues, who found no significant differences in the TDI score between PD patients with and without OHs (16.0 ± 4.8 vs. 18.1 ± 7.6; *p* = 0.294) [40], although TDI mean scores were indicative of hyposmia in both groups. Both Hirsh and colleagues, as well as Landis and Burkhard, assessed the olfactory function of their patients with phantosmia using the Sniffin’ Sticks test [25,27]. Their findings revealed one patient with anosmia, two with hyposmia, and one with normal olfactory function [25,27]. The UPSIT was used by Bannier and colleagues, who identified olfactory dysfunction in eight out of nine PD patients with OHs, and they did not observe any difference between patients with and without OHs [19]. Mehanna and colleagues assessed olfactory function in their large sample using the UPSIT and found that 84% of PD patients with phantosmia exhibited severe microsmia or anosmia [33].

### 3.5. Associated Hallucinations

While these data have not been exhaustively detailed, several studies have indicated the concurrent presence of other types of hallucinations alongside phantosmia [18,19,20,21,22,23,24,26,28,29,30,33,35,36,37,39,40,41,42,43,44]. Importantly, in the study by Solla and colleagues, nine patients with OHs (56.3%) also reported visual hallucinations and auditory hallucinations, and four (25%) had gustatory hallucinations. Among the 25 patients with OHs described by Mehanna and colleagues, 8 (32%) also had visual hallucinations, 6 (24%) had auditory hallucinations, and 3 (12%) had tactile hallucinations [33]. Bannier and colleagues identified two out of nine (22.2%) PD patients with phantosmia who had visual hallucinations and three out of nine (33.3%) who had both visual hallucinations and auditory hallucinations [19].

### 3.6. Impact on Quality of Life

Only five studies have investigated the impact of OHs on the quality of life of PD patients. These studies showed that phantosmia can variably affect daily activities. Specifically, Bannier and colleagues noted that for their nine patients, the quality of life was only minimally impacted since they recognized the hallucinatory nature of the phenomenon and did not consider OHs frightening [19]. Conversely, McAuley and Gregory documented one patient exhibiting obsessive behavior due to phantosmia, another suffering from sleep disturbances linked to olfactory misperception, and yet another who was notably bothered by OHs [32]. On a similar note, Arnulf reported that phantosmia led to distress and family issues due to the aggressive behavior exhibited by the affected patient [18]. Meanwhile, Tousi and Frankel noted that their patient was irritated by the sensation of a dry throat, which they attributed to the perceived smoke [42].

### 3.7. Treatment of OHs

Since none of the studies in the literature were pharmacological trials focusing on the management of phantosmia in PD patients, the information related to the treatment of OHs was anecdotal, primarily arising from individual case descriptions [25,27,32,42]. Generally, the most common pharmacological interventions included (i) the adjustment of antiparkinsonian therapy through a gradual reduction in the levodopa equivalent daily dose and (ii) the introduction of atypical neuroleptics. The outcomes reported in these studies were often positive, with an improvement or disappearance of phantosmia. None of the patients included in this review suffered from phantosmia during motor fluctuations.

## 4. Discussion

This review focuses on the presence of OHs or phantosmia in PD patients, a neglected type of hallucination that has received little attention in existing research, even though they represent a clinical issue in the daily management of parkinsonian patients [45,46]. OHs have been frequently studied in the context of psychiatric disorders or neurological conditions due to epilepsy or COVID-19 infection, while their features in PD are less understood [13,14,15]. Among non-visual hallucinations, OHs may frequently occur in PD patients, but they are often overlooked in clinical practice [47]. Moreover, clinicians should always check for phantosmia during the disease’s clinical course, not just at the baseline assessment, as longitudinal studies have identified phantosmia in initially unaffected PD patients [32,48,49]. In this review, we specifically focus our attention on the frequency of OHs in the PD population and the gender distribution; the features of phantosmia (pleasant vs. unpleasant); the assessment methods used by the authors in order to evaluate OHs in PD patients; the olfactory function of the PD patients with phantosmia; the associated hallucinations that might coexist with OHs in the same subject; the impact of phantosmia on the quality of life of PD patients; and the treatment used to manage phantosmia. Due to the significant heterogeneity among the included studies in terms of study design, we were unable to combine those findings. For instance, regarding cognitive impairment, only two studies assessed cognitive ability in patients with phantosmia, but they used two different and non-comparable tests: Solla and colleagues performed the MoCA [40], while Bannier and colleagues used the MMSE [19].

Indeed, our review revealed that OHs in PD patients seem more common than previously recognized, with reported prevalence rates ranging from 0.5% to 18.2%. These variations in frequency distribution might be attributed to heterogeneity in study populations, the inclusion and exclusion criteria for study participation, the assessment tools used to identify OHs, and the duration of the investigation [50,51].

Phantosmia has also been described in other neurological conditions; however, comprehensive studies are still lacking, as is the case in PD. For instance, Scarmeas and colleagues found that 2% of their patients with Alzheimer’s disease suffered from OHs at baseline, whereas during the follow-up evaluation, this percentage increased to 4.4% of their sample [52]. Another study confirmed the infrequent occurrence of phantosmia in Alzheimer’s disease patients compared to the presence of auditory or visual hallucinations [53]. Focusing on neuropsychiatric disorders, Schutte and colleagues reported OHs in 53% of their 137 patients with major psychiatric disorders, a heterogeneous group that included schizophrenia spectrum disorder, mood disorders, post-traumatic stress disorder, and borderline personality disorder [45]. When comparing this group with 47 patients with neurodegenerative disorders (such as PD, dementia with Lewy bodies, and Alzheimer’s disease), the authors found that 14.9% of the patients with neurodegenerative diseases suffered from phantosmia [45]. Although the association between olfactory dysfunction and RBD has already been established [54], we did not find any studies reporting phantosmia in RBD patients.

The gender distribution of phantosmia presented contrasting findings as well. While some studies, like those by Solla and colleagues and Bannier and colleagues, reported a female predominance [19,40], others, such as Mehanna’s study, revealed an equal or even male-centric distribution [33]. Noteworthy, the study by Solla and colleagues showed that female sex was a clinical variable predicting the presence of OHs in PD patients [40]. These data reinforced the putative central role of sex differences in the development of NMSs in PD patients, with particular references to olfactory symptoms [55,56,57]. Future research on phantosmia should explore the potential influence of sex on the development of OHs in PD patients.

The vast array of scents described by PD patients affected by phantosmia, ranging from pleasant aromas of flowers and fruits to foul smells of garbage or burning, confirmed the important subjective nature of OHs and raised several questions about the pathophysiological origins of these symptoms. Since most of the included studies did not accurately report the percentage of patients suffering from one specific odor compared to another, it was not possible to conduct a comprehensive comparison or to identify trends across the entire population. While quantitative olfactory dysfunctions, such as anosmia and parosmia, may only partially account for OHs in PD patients, other neural mechanisms warrant exploration [19,40]. Indeed, the studies conducted by Bannier [19] and Solla [40] revealed no notable distinctions in OT, OD, and OI between PD patients with phantosmia and those without it. Consequently, it appears that OHs in PD patients are only partially connected to the quantifiable olfactory impairments that result in hyposmia or anosmia. Analogous to what has been recently postulated for visual hallucinations, a model of aberrant hierarchical predictive processing in the olfactory bulb and in the anterior olfactory nucleus might explain the occurrence of phantosmia in PD [58]. These hypotheses gain significance when considering that our review highlighted a high frequency of co-occurrence between OHs and other types of hallucinations, especially visual ones. Notably, the common association highlighted in this review between OHs and either visual hallucinations and/or auditory hallucinations suggests that the pathophysiological mechanisms behind these phenomena might overlap [59].

One crucial point that emerged from this systematic search of the literature is the lack of a specific and validated tool for the assessment of phantosmia in PD patients. Most of the studies included in this review used semi-structured interviews or other structured questionnaires designed primarily for an initial evaluation of psychosis in PD [16,17,18,19,20,21,22,23,24,25,26,27,28,29,30,31,32,33,34,35,36,37,38,39,40,41,42,43,44]. As already mentioned, the heterogeneity in assessment methods may contribute to the variation in reported prevalence. Indeed, none of the studies has systematically assessed this issue, and we based our information on data described in a few studies [18,19,25,32]. A necessary next step toward better understanding OHs in PD patients is the creation and validation of a specific questionnaire, which should also address the impact of phantosmia on quality of life. Within this context, we have highlighted the most relevant questions to be included in a specific questionnaire for phantosmia in Table 2.

With no pharmacological trials focusing specifically on phantosmia in PD, treatment strategies are largely based on case descriptions [25,27,32] and the general management of psychosis in PD [59,60]. The approach to managing OHs aligns with the treatment of other psychotic symptoms. The first measure involves looking for recent triggers (i.e., symptomatic or occult infection) or changes in medication. The subsequent step is to adjust the patient’s current daily therapy, with a particular focus on medications that could exacerbate the hallucinations. Following that, one should consider specific treatments [59,60].

Although none of the studies reported an association between phantosmia and motor fluctuations, this point deserves deeper consideration. Indeed, previous studies have already highlighted a lack of association between LEDD and OHs [40]. However, none of them have investigated the possibility that OHs may fluctuate like other NMSs in the phenomenon known as non-motor fluctuations (NMFs) [61]. NMFs rely on dopaminergic and non-dopaminergic mechanisms and are often overlooked in clinical practice [61]. Further studies are needed to better address the possibility that the presence of phantosmia may fluctuate during the course of the disease.

This review, while providing valuable insights, was not without its limitations. Firstly, the studies chosen for analysis exhibited significant heterogeneity. This was evident in the diverse clinical features of the patients studied, including their cognitive status, disease duration, and clinical severity. Furthermore, the inclusion and exclusion criteria for participation in these studies varied widely. The methodology each study adopted to detect OHs also differed, introducing further complexity. All these factors made direct comparisons between the studies quite challenging, and we were not able to identify specific trends for the entire population. The lack of comprehensive data should serve as a stimulus for clinicians and researchers to include phantosmia in their research agendas. Another area of concern arises from the inherent variability in PD itself. Different clinical subtypes and varying disease stages of PD might manifest with different prevalence rates of OHs. This suggests that there could be variations not only in the number of patients exhibiting phantosmia but also in the specific characteristics of the phantosmia they experience. Moreover, the absence of longitudinal data on the progression of OHs limits a comprehensive understanding of these symptoms in PD [62].

In conclusion, this systematic review sheds light on the complex phenomenology of OHs in PD and emphasizes the need for comprehensive assessment and management of phantosmia in clinical practice. Future efforts should prioritize the creation and validation of standardized assessment tools. Further research is needed to explore the underlying mechanisms and develop targeted interventions for these distressing symptoms.

## Figures and Tables

**Table 1:** Information on the 29 studies included in this systematic review.

Ref	Authors	Year of Publication	Study Design	Sample Size	Number of Patients with OHs (%)
[16]	Aarsland et al.	2001	cross-sectional	83 PD without dementia and 48 PDD	1 (1%) PD and 3 PDD (6%)
[17]	Amar et al.	2014	cross-sectional	40 PD	1 (2.5%)
[18]	Arnulf et al.	2000	case report	1 PD	1
[19]	Bannier et al.	2012	cross-sectional	87 PD	9 (10%)
[20]	Barrett et al.	2017	cross-sectional	101 PD	4 (4%)
[21]	Chou et al.	2005	baseline data from 2 studies on PD patients with drug-induced psychosis	160 PD	25 (16%)
[22]	Factor et al.	2014	cross-sectional	144 PD	7 (4.9%)
[23]	Fenelon et al.	2010	cross-sectional	116 PD	13 (11%)
[24]	Gu et al.	2022	cross-sectional	278 PD	4 (5.2%)
[25]	Hirsch et al.	2009	case report	1 PD	1
	Burkhard and Landis	2009	case report	1 PD	1
[26]	Kulick et al.	2018	cross-sectional	199 PD	11 (5.5%)
[27]	Landis and Burkhard	2008	case series	1 PD	1
				1 PD	1
[28]	Lenka et al.	2017	cross-sectional	51 PD	1 (1.9%)
[29]	Mack et al.	2012	cross-sectional	250 PD	5 (2%)
[30]	Marques et al.	2022	cross-sectional	62 PD	5 (8%)
[31]	Marsh et al.	2004	cross-sectional	116 PD	3 (2.6%)
[32]	McAuley and Gregory	2012	cross-sectional plus case description	188 PD	4 (2.1%) at baseline and 2 (1%) at follow-up
[33]	Mehanna et al.	2022	cross-sectional	137 PD	25 (18.2%)
[34]	Muller et al.	2018	cross-sectional	163 PD	15 (9%)
[35]	Omoto et al.	2020	cross-sectional	100 PD	2 (2%)
[36]	Pagonabarraga et al.	2015	baseline data from a longitudinal study	50 PD	2 (4%)
[37]	Papapetropoulos et al.	2008	cross-sectional	70 PD	5 (7.1%)
[38]	Rai et al.	2015	cross-sectional	126 PD	2 (1.6%)
[39]	Shine et al.	2014	cross-sectional	197 PD	18 (9%)
[40]	Solla et al.	2021	cross-sectional	141 PD	16 (11.3%)
[41]	Svetel et al.	2012	cross-sectional	180 PD	1 (0.5%)
[42]	Tousi and Frankel	2004	case report	1 PD	1
[43]	Whitehead et al.	2008	case series	50 PD	3 (6%)
[44]	Zhang et al.	2022	cross-sectional	149	8.1%

**Table 2:** Questionnaire for the identification and evaluation of olfactory hallucinations in Parkinson’s disease patients.

Question Category	Question	Scoring
Symptom Assessment	Have you experienced any unusual or abnormal smells that others do not seem to notice? (Yes/No)	Yes: 1 point; No: 0 points
Symptom Description	Describe whether these smells are pleasant or unpleasant.	N/A
	Describe the nature of these smells (e.g., foul, sweet, burning, chemical).	N/A
Frequency	How often do you experience these smells? (Daily, Weekly, Monthly)	Daily: 3 points; Weekly: 2 points; Monthly: 1 point; Never: 0 points
Onset	How long have you noticed these smells?	<6 months: 1 point; 6–12 months: 2 points; >12 months: 3 points
Consistency	Do these smells occur at specific times, or are they random? (Specific Times/Random)	Specific Times: 2 points; Random: 1 point
Correlation with PD	Do you notice these smells more during certain states of your PD (e.g., ON/OFF states)? (Yes/No)	Yes: 2 points; No: 0 points
State Description	If yes, please describe these states and their relation to the smells.	N/A
Impact on Daily Life	Do these smells affect your eating habits? (Yes/No)	Yes: 1 point; No: 0 points
	Do these smells interfere with daily activities? (Yes/No)	Yes: 1 point; No: 0 points
Distress Level	Rate the distress caused by these smells on a scale of 0 to 10.	0–10 scale
Associated Symptoms	Do you experience any other symptoms along with the smells? (Please describe, e.g., headaches, dizziness, pain, sweating.)	N/A
Treatment and Management	Have you noticed any factors or conditions that seem to trigger or worsen these unusual smells? (Please describe.)	N/A
	Are there any activities, treatments, or conditions that appear to alleviate or reduce these smells? (Please describe.)	N/A
	Are you currently receiving any treatment specifically for these smells?	N/A
Additional Comments	Please provide any additional information or comments regarding your experience with phantosmia.	N/A

N/A: not available.

## Data Availability

The datasets analyzed for this systematic review are available in the cited articles and publications. References for all articles included in this review can be found in the bibliography. No original datasets were generated or analyzed for this review. Readers with further inquiries about specific datasets should contact the original authors of the cited studies.

## Abbreviations

eSAPS-PD	Enhanced Scale for Assessment of Positive Symptoms in Parkinson’s Disease
NMSs	non-motor symptoms
NMFs	non-motor fluctuations
NPI	Neuropsychiatric Inventory
OHs	olfactory hallucinations
PD	Parkinson’s disease
PSAS	Psycho-Sensory hAllucinations Scale
PsycH-Q	Psychosis and Hallucinations Questionnaire
RBD	rapid eye movement sleep behavior disorder
SAPS	Schedule for Assessment of Positive Symptoms
TDI	threshold–discrimination–identification
UM-PDHQ	University of Miami Parkinson’s disease Hallucinations Questionnaire
UPSIT	University of Pennsylvania Smell Identification Test
